# Mutant Screen Distinguishes between Residues Necessary for Light-Signal Perception and Signal Transfer by Phytochrome B

**DOI:** 10.1371/journal.pgen.1000158

**Published:** 2008-08-15

**Authors:** Yoshito Oka, Tomonao Matsushita, Nobuyoshi Mochizuki, Peter H. Quail, Akira Nagatani

**Affiliations:** 1 Department of Biology, Graduate School of Science, Kyoto University, Kyoto, Japan; 2 Department of Plant and Microbial Biology, University of California Berkeley, Berkeley, California, United States of America; 3 Plant Gene Expression Center, U.S. Department of Agriculture, Albany, California, United States of America; 4 Graduate School of Agriculture, Kyushu University, Fukuoka, Japan; 5 Organization for the Promotion of Advanced Research, Kyushu University, Fukuoka, Japan; The University of North Carolina at Chapel Hill, United States of America

## Abstract

The phytochromes (phyA to phyE) are a major plant photoreceptor family that regulate a diversity of developmental processes in response to light. The N-terminal 651–amino acid domain of phyB (N651), which binds an open tetrapyrrole chromophore, acts to perceive and transduce regulatory light signals in the cell nucleus. The N651 domain comprises several subdomains: the N-terminal extension, the Per/Arnt/Sim (PAS)-like subdomain (PLD), the cGMP phosphodiesterase/adenyl cyclase/FhlA (GAF) subdomain, and the phytochrome (PHY) subdomain. To define functional roles for these subdomains, we mutagenized an *Arabidopsis thaliana* line expressing N651 fused in tandem to green fluorescent protein, β-glucuronidase, and a nuclear localization signal. A large-scale screen for long hypocotyl mutants identified 14 novel intragenic missense mutations in the N651 moiety. These new mutations, along with eight previously identified mutations, were distributed throughout N651, indicating that each subdomain has an important function. *In vitro* analysis of the spectral properties of these mutants enabled them to be classified into two principal classes: light-signal perception mutants (those with defective spectral activity), and signaling mutants (those normal in light perception but defective in intracellular signal transfer). Most spectral mutants were found in the GAF and PHY subdomains. On the other hand, the signaling mutants tend to be located in the N-terminal extension and PLD. These observations indicate that the N-terminal extension and PLD are mainly involved in signal transfer, but that the C-terminal GAF and PHY subdomains are responsible for light perception. Among the signaling mutants, R110Q, G111D, G112D, and R325K were particularly interesting. Alignment with the recently described three-dimensional structure of the PAS-GAF domain of a bacterial phytochrome suggests that these four mutations reside in the vicinity of the phytochrome light-sensing knot.

## Author Summary

Adapting to the light environment, plants have evolved several photoreceptors, of which the phytochromes are specialized in perceiving the red and far-red light region of the spectrum. Although phytochrome was first discovered in plants, the phytochrome species are present in several organisms, including bacteria. The mechanisms by which phytochromes transduce light signals to downstream components are most well studied in plants. Upon light activation, phytochromes translocate from the cytoplasm into nucleus and regulate the gene expression network through interaction with nuclear transcription factors. The phytochrome molecule can be divided into two major domains: the N-terminal moiety, which is responsible for the light perception, and the C-terminal moiety. Although the C-terminal moiety was though to be involved in signal transduction, it has recently been shown that the N-terminal moiety has a role not only in the light perception, but also in light signal transfer to the downstream network. However, no signaling motifs have been found in the N-terminal moiety. In this study, we analyzed intragenic mutations derived from a genetic screen and found a cluster of residues necessary for signal transduction in a small region neighboring the light-sensing chromophore moiety on the three-dimensional structure. This is an important step towards understanding how a major plant photoreceptor, phytochrome, intramolecularly processes the light signal to trigger diverse physiological responses.

## Introduction

To adapt to fluctuating environmental conditions, plants obtain and interpret information from light. These light sensing processes utilize at least three classes of photoreceptors [1]–[3] of which phytochromes are well characterized with respect to molecular structure and biological function. Phytochromes are unique pigments whose function is mediated through photoreversible conformational changes between two spectrally distinct forms: an inactive red-light (R)-absorbing form (Pr) and an active far-red-light (FR)-absorbing form (Pfr). R converts Pr to Pfr, and FR converts Pfr back to Pr. In addition, Pfr is gradually converted back to Pr in darkness by a thermally driven process called “dark reversion”. In *Arabidopsis* the phytochrome family consists of five members [4]. Two members of the family, phytochrome A (phyA) and B (phyB) are the most important in seedling development. PhyA and phyB have different photosensory specificities. PhyA mediates de-etiolation under continuous FR (cFR), whereas phyB mediates de-etiolation under continuous R (cR) [5].

Phytochromes, which are soluble proteins, are synthesized in the Pr form and reside in the cytoplasm in darkness. Upon light activation, phytochromes translocate to the nucleus [6]–[9] where they regulate gene expression [10]–[12]. Phytochromes interact with nuclear basic helix-loop-helix proteins such as PIF3 in a light-dependent manner [13]–[15]. These interactions are thought to induce alterations in the expression of target genes [16],[17].

Phytochromes in solution exist as dimers of approximately 120 kD subunits, each of which binds a single open tetrapyrrole chromophore responsible for the absorption of visible light. Each phytochrome monomer consists of a chromophore-bearing N-terminal moiety of about 70 kD and a C-terminal moiety of about 55 kD. The N-terminal moiety is highly conserved among members of the phytochrome family. The N-terminal moiety alone can bind the chromophore and show photoreversible conformational changes. On the other hand, the C-terminal moiety is required for dimerization [18] and nuclear localization [19]. Although the C-terminal moiety had long been presumed to transduce the signal to downstream components, we have shown that the N-terminal moiety of phyB alone can transduce the signal in the nucleus in response to light stimuli [20]. The data indicate therefore, that the N-terminal moiety has not only a light perception function but also a signal transferring function.

Although phytochromes were originally discovered in plants, recent analyses have demonstrated that phytochrome-related molecules are found in various bacteria [21]. Based on sequence analysis, four domains are recognized in the N-terminal moiety of phytochromes: the N-terminal extension, the N-terminal Per/Arnt/Sim (PAS)-like domain (PLD), the cGMP phosphodiesterase/adenyl cyclase/FhlA domain (GAF), and the phytochrome domain (PHY) [21]. The N-terminal extension is found in higher plant phytochromes but not in bacteriophytochromes. GAF has bilin lyase activity and covalently binds the chromophore [22]. PHY stabilizes Pfr [23]. Although the crystal structure of plant phytochromes has not been determined yet, that of the PAS-GAF domain of *Deinococcus radiodurans* bacteriophytochome (*Dr*CBD) has been determined [24]. Interestingly, an unusual three dimensional structure, designated the light sensing knot [24], is found between the PAS and GAF domains in *Dr*CBD.

To identify regions of the protein important for signal transduction by phytochromes, several deletion derivatives have been examined for their biological activities [23], [25]–[29]. According to those studies, a phyB derivative that lacks the N-terminal 103 amino acid extension exhibits reduced but significant biological activity [29]. Similarly, the PHY subdomain is dispensable for the signaling activity [23]. Hence, the core region of phyB responsible for signal transduction activity can be narrowed down to the region composed of PLD and GAF. However, critical amino acid residues necessary for signaling have not been identified.

Mutational analyses have been adopted for the study of the phytochrome signal transduction mechanism. Several amino acid substitutions within phytochrome molecules have been identified that reduce the biological activity of the molecule without affecting either the amount of protein accumulation or the photochemical properties of the protein. Although this kind of mutational analysis led to identification of the Quail-box, which resides in the C-terminal moiety [30], it has been later shown that some of the mutations in this region impair the subcellular dynamics of phytochromes [8],[20]. On the other hand, as the N-terminal moiety retains dual functions (a light perception and a signal transferring function) the amino acid substitutions, which reduce the biological activity, within the N-terminal moiety may be expected to fall into two classes: (1) one consisting of those that are defective in photoperception and/or the maintenance of the active Pfr form, and (2) the other containing those that are normal in photoperception and the maintenance of active Pfr form, but defective in regulatory activity.

**Figure 1:**
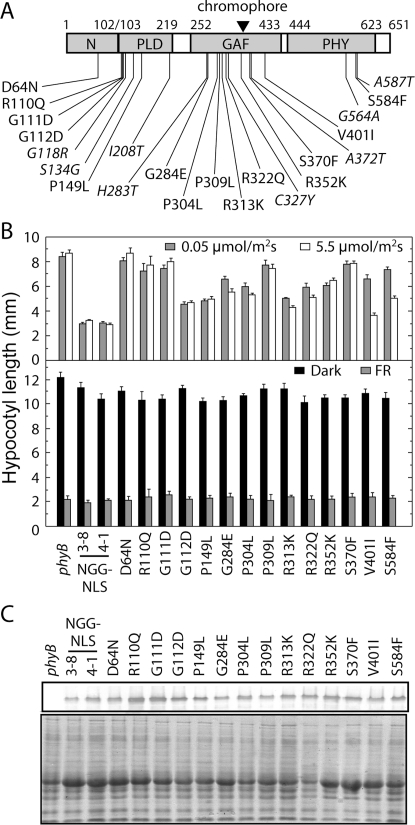
Hypocotyl Phenotypes of N651-GUS-NLS Mutants Carrying Missense Mutations. (A) Locations of missense mutations found in the present (plain) and previous (italic) studies. For details, see Table 1. PLD, GAF and PHY were delimited as amino acid residues 103–219, 252–433 and 444–623, respectively, according to a sequence-based domain database, Pfam version 20.0 (http://www.sanger.ac.uk/Software/Pfam). The N-terminal extension was defined as amino acid residues 1–102. The closed triangle represents the chromophore binding site. (B) Hypocotyl lengths of mutants grown under different light conditions. For the hypocotyl measurement, plants were grown under weak cR (0.05 µmol m^−2^ sec^−1^) (shaded, upper panel), strong cR (5.5 µmol m^−2^ sec^−1^) (open, upper panel), cFR (10 µmol m^−2^ sec^−1^) (shaded, lower panel) or in darkness (closed, lower panel). The mean±SE (*n* = 25) is shown. (C) Immunoblot detection of the N651G-GUS-NLS proteins. For detection, 50 µg of total protein was loaded in each lane, blotted onto nitrocellulose membrane after SDS-PAGE, and probed with an anti-GFP monoclonal antibody (SIGMA) (upper panel). To confirm protein loading amount, the same samples were subjected to Coomassie Brilliant Blue (CBB) staining (lower panel).

Of the above two classes, the latter class of mutations is thought to directly disrupt the signal transfer to components downstream of phyB. Although altogether 8 mutations have been reported within the N-terminal moiety of phyB [23], [31]–[34], none have been fully investigated. This may be because the signal transferring function of N-terminal moiety had not been established until the recent evidence that our engineered N-terminal moiety of phyB can complement the *phyB* mutation [20]. In addition, the number of mutations reported within the N-terminal moiety is too few and the distribution throughout the N-terminal moiety is too disperse to indicate regions important for signal transduction of phyB (Figure 1A, Table 1).

**Table 1:** Summary of the phyB Mutations.

Mutation	Domain	Hypocotyl Phenotype^a^	Spectral Deficiency			Reference
			Chromophore Incorporation	Difference Spectrum	Dark Reversion	
D64N	N^b^	+++	−	−	−	this work
R110Q	PLD	+++	−	−	−	this work
G111D	PLD	+++	−	−	−	this work
G112D	PLD	+	−	−	−	this work
G118R	PLD	nd^c^	++	nd	nd	[31],[32]
S134G	PLD	nd	++	nd	nd	[32]
P149L	PLD	+	−	−	−	this work
I208T	PLD	nd	−	−	−	[32]
H283T	GAF	nd	−	−	+	[34]
G284E	GAF	++	+	+	nd	this work
P304L	GAF	++	−	−	−	this work
P309L	GAF	+++	+	+	nd	this work
R313K	GAF	+	−	−	+	this work
R322Q	GAF	++	−	+	++	this work
C327Y	GAF	nd	−	−	+	[31]
R352K	GAF	++	−	−	−	this work
S370F	GAF	+++	++	+	nd	this work
A372T	GAF	nd	−	+	++	[31]
V401I	GAF	++	−	−	+	this work
G564A	PHY	nd	−	−	+	[23],[33]
S584F	PHY	+++	−	+	++	this work
A587T	PHY	nd	−	−	+	[31]

a Long hypocotyl phenotype under cR (0.05 µmol m^−2^ sec^−1^).

b N-terminal extension.

c nd, not determined.

Here, to first identify the critical amino acid residues necessary for signal transfer, we performed a large scale genetic screen for long hypocotyl mutants under dim cR. In this screen, we mutagenized *Arabidopsis thaliana* expressing the engineered N-terminal moiety of phyB in order to focus on this moiety. Our data identify two classes of residues with functionally distinct roles, respectively, in photosensory perception and signal propagation to downstream targets.

## Results

### Identification of New Missense Mutations within the N-Terminal Moiety of phyB

The N-terminal 651 amino acid fragment of phyB (N651), fused in tandem to green fluorescent protein (GFP), β-glucuronidase and a nuclear localization signal (NLS) (N651G-GUS-NLS), is fully functional in all phyB responses examined, and exhibits hypersensitivity to cR for various phyB responses [20] except for root greening under red light [35]. To identify amino acid residues that are important for N651 function, an *Arabidopsis* line expressing N651G-GUS-NLS in the *phyB* mutant background was mutagenized with ethyl methanesulfonate (EMS), and the M2 seedlings were screened for the long hypocotyl phenotype under weak cR (0.05 µmol m^−2^ sec^−1^).

At least 1,000,000 M2 seedlings derived from 200,000 M1 plants were subjected to screening. Putative mutant lines were examined further in the M3 generation. GFP fluorescence was severely reduced in more than 90% of these lines. The lines in which GFP fluorescence was not reduced were further examined with respect to the hypocotyl phenotype under cFR. We selected 69 lines that showed the long hypocotyl phenotype only under cR. These 69 lines were crossed with the *phyB* mutant. Subsequent segregation analysis in the F2 generation revealed that 19 of them were linked to the *N651G-GUS-NLS* gene, indicating that they were intragenic mutants. Sequence analysis of these 19 lines revealed an amino acid substitution within the *N651* moiety in each line. These 19 lines yielded 14 distinct substitutions representing, therefore, 14 different variants of the *N651* gene (Figure 1A, Table 1). None of these mutations has been reported previously [30]–[34]. We confirmed that no mutations were found in the *GFP-GUS-NLS* moiety in these lines. GFP fluorescence was observed exclusively in the nucleus in each line (data not shown), verifying their expected constitutive nuclear localization.

The hypocotyl lengths of these lines compared to the *phyB* null mutant and the parental N651G-GUS-NLS 4-1 and N651G-GUS-NLS 3–8 lines, under two intensities of cR, are shown in Figure 1B. As we described previously [20],[23], the lower of these two intensities of cR (0.05 µmol m^−2^ sec^−1^) is already saturating for inhibition of hypocotyl elongation in these parental N651G-GUS-NLS lines, so that no difference in hypocotyl length between the two intensities was observed for these two lines. Each of the mutant lines, on the other hand, exhibited a long hypocotyl phenotype, to varying degrees compared to the N651G-GUS-NLS lines, with some displaying cR-intensity responsiveness, and others not. The hypocotyls of D64N, R110Q, G111D, P309L, and S370F lines were almost as long as those of the *phyB* mutant under both intensities, indicating severe or complete loss of phyB activity. The remaining nine variants showed an intermediate hypocotyl-length phenotype between the *phyB* parent and the N651G-GUS-NLS transgenic rescue lines. Of these, six (G248E, P304L, R313K, R322Q, V401L and S584F) showed a greater or lesser degree of reduced responsiveness to the lower compared to the higher cR intensity, whereas the remaining three (G112D, P149L and R352K) did not show such a difference in hypocotyl responsiveness to the cR intensity. We confirmed that the long hypocotyl phenotype was observed neither under cFR nor in darkness (Figure 1B).

Immunoblot blot analysis of light grown seedlings showed that the mutant-variant lines contain levels of the phyB fusion-protein similar to or higher than the parental N651G-GUS-NLS 4-1 line, in most cases (Figure 1C). Although the levels were reduced in some lines, they were still higher than that in another N651G-GUS-NLS line, 3–8 (Figure 1C), in which the full response to cR was observed (Figure 1B). Concordant results were obtained from measuring GUS activity in these lines (data not shown). These data indicate that the reduced responsiveness to cR is due to reduced intrinsic activity of the mutant phyB rather than reduced levels of expression.

### Effects of Missense Mutations on Chromophore Incorporation

**Figure 2:**
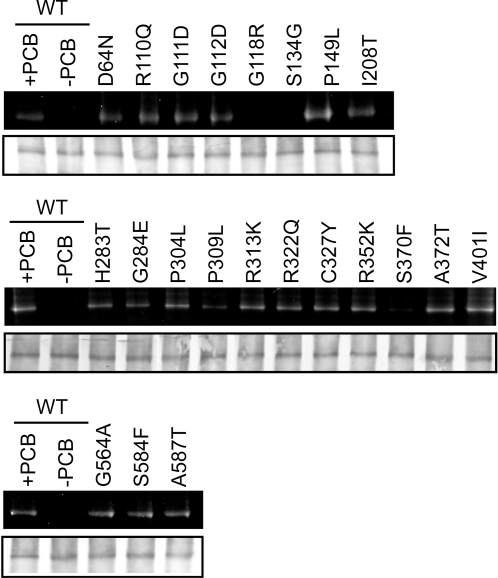
Chromophore Ligation to Mutant N651 Fragments. Results of zinc-blot (upper panels) and immunoblot (lower panels) analyses are shown. Crude extracts from *Escherichia coli* expressing the mutant N651 fragments were incubated with 5 µM PCB and separated by SDS-PAGE [23],[37]. Immunoblot detection was performed using antiserum against chitin binding domain (New England Biolabs).

In addition to the 14 mutations described above, 8 missense mutations within the N651 moiety that reduce the function of phyB have been reported previously [23], [31]–[34]. These 22 missense mutations in the N-terminal moiety of phyB are detailed in Figure 1A and Table 1. The spectral characteristics had been examined for only two of these mutations [23],[36] prompting us to examine the entire cohort for spectral integrity. Spectrally active phyB derivatives were reconstituted *in vitro* using phycocyanobillin (PCB) as the chromophore [37]. Wild type and mutated N651 fragments fused to intein and chitin binding domain (CBD) were expressed in *E. coli* and subjected to chromophore incorporation analysis [23]. The crude extracts from *E.coli* were mixed with phycocyanobilin (PCB) and examined by the Zn blot assay (Figure 2). The results showed that 17 mutants displayed normal PCB incorporation. Of the remaining 5 mutants, PCB incorporation was not detected in G118R and S134G, was reduced in G284E and P309L, and was markedly reduced in S370F.

### Effects of Missense Mutations on Pr-Pfr Difference Spectra

**Figure 3 pgen-1000158-g003:**
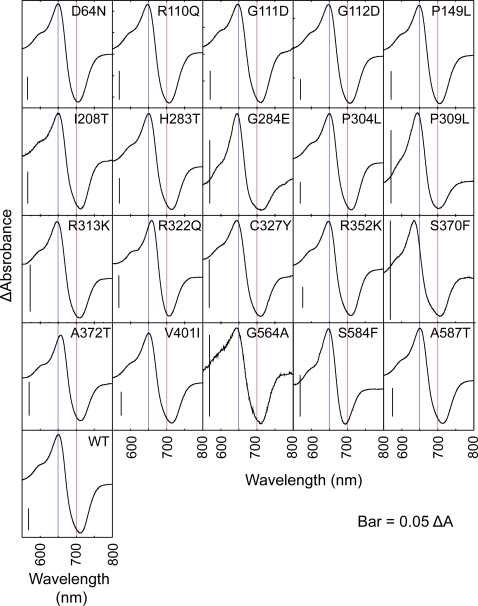
Pr-Pfr Difference Spectra of Mutant N651 Fragments. The mutated holoproteins were prepared as for Figure 2 and subjected to spectrophotometry. Blue and red lines indicate 650 and 700 nm, respectively.

We examined whether the mutations affected the spectral properties of N651. Twenty mutants that allowed chromophore incorporation (Table 1) were tested for the Pr-Pfr difference spectrum (Figure 3). The spectrum for the wild type N651 fragment exhibited an absorption maximum around 650 nm and minimum around 710 nm as previously described [23]. Of these 20 mutants, 14 mutants exhibited normal difference spectra. The remaining 6 mutants, G284E, P309L, R322Q, S370F, A372T and S584F, exhibited abnormal difference spectra. The G284E and P309L mutants exhibited a bleached spectrum in which the trough in the far-red region was much shallower compared with the peak in the red region. The S584F mutant exhibited a similar defect but to a lesser extent. In addition, a substantial blue-shift of the difference spectrum minimum was observed in this mutant. In R322Q and A372T, a red-shift of the difference spectrum maximum was observed. Conversely, a blue-shift of the difference spectrum maximum was observed in S370F.

### Effects of Missense Mutations on Dark Reversion Rates

**Figure 4:**
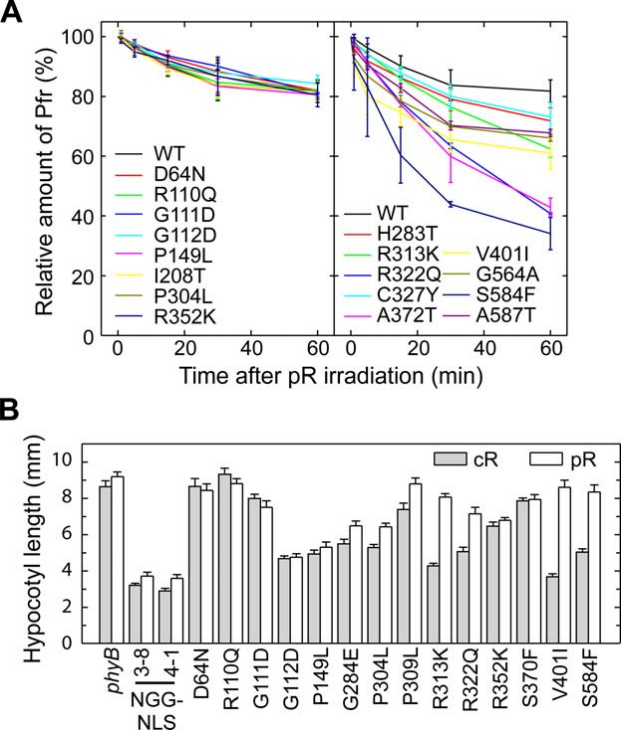
Dark Reversion Rates in the Mutant N651 Fragments. (A) *In vitro* dark reversion rates measured by spectrophotometry. Holoproteins were converted to Pfr by irradiation with saturating R (89 µmol m^−2^ sec^−1^) for 10 min, and then dark reversion from Pfr to Pr was monitored with a spectrophotometer. The level of Pfr at the beginning of the measurement was set to 100%. The incubation temperature was 22°C. Each value represents the mean of three independent measurements. (B) Hypocotyl responses in mutant lines to intermittent pR at 4 hr intervals. Plants were grown under intermittent pR (55.2 µmol m^−2^ sec^−1^ for 5 min) (open) or under cR (2.3 µmol m^−2^ sec^−1^) (shaded) for 5 days. Data are the mean±SE (*n* = 25).

The Pfr form of phytochrome is thermally unstable, and it spontaneously converts back to Pr in darkness by a process called ‘dark reversion’. This dark reversion is an important process to regulate the level of Pfr *in vivo*. Hence, we compared the dark reversion rates in the wild type and the N651 mutants (Figure 4A). Those mutants that were severely deficient in chromophore incorporation (G118R, S134G, G284E, P309L and S370F) were excluded from this analysis. As has been reported previously, the wild type N651 exhibited a relatively slow dark reversion rate, with more than 80% remaining as Pfr 1 hr after a pulse of R (pR). Eight out of the 17 mutants exhibited similar dark reversion rates to that in wild type N651 (Figure 4A, Table 1). The other 9 mutants, to various extents, exhibited an increase in the dark reversion rate. Three mutants in particular, S584F, R322Q and A372T, exhibited a very fast dark reversion rate with only 40% remaining as Pfr 1 hr after pR.

The hypocotyl response to intermittent pR depends very much on the stability of Pfr in darkness [23]. Hence, we examined how the mutant plants responded to cR and pR (Figure 4B). This was done only in the 14 mutants that were obtained in the present study (Table 1). As expected, S584F and R322Q, in which the dark reversion rates were very fast *in vitro* (Figure 4A), exhibited reduced responses to pR. Similar differences were observed in R313K and V401, both of which exhibited relatively fast dark reversion rates. In addition, we observed smaller but significant differences in G284E, P304L and P309L. Of these, the dark reversion rate was not measured in G284E and P309L because severe reduction in chromophore incorporation (Figure 2) and bleached difference spectra (Figure 3) were observed. Exceptionally, P304L did not exhibit any significant phenotype with respect to spectral properties *in vitro*.

### Regulatory Activity of Full-length phyB Mutant Variants

**Figure 5:**
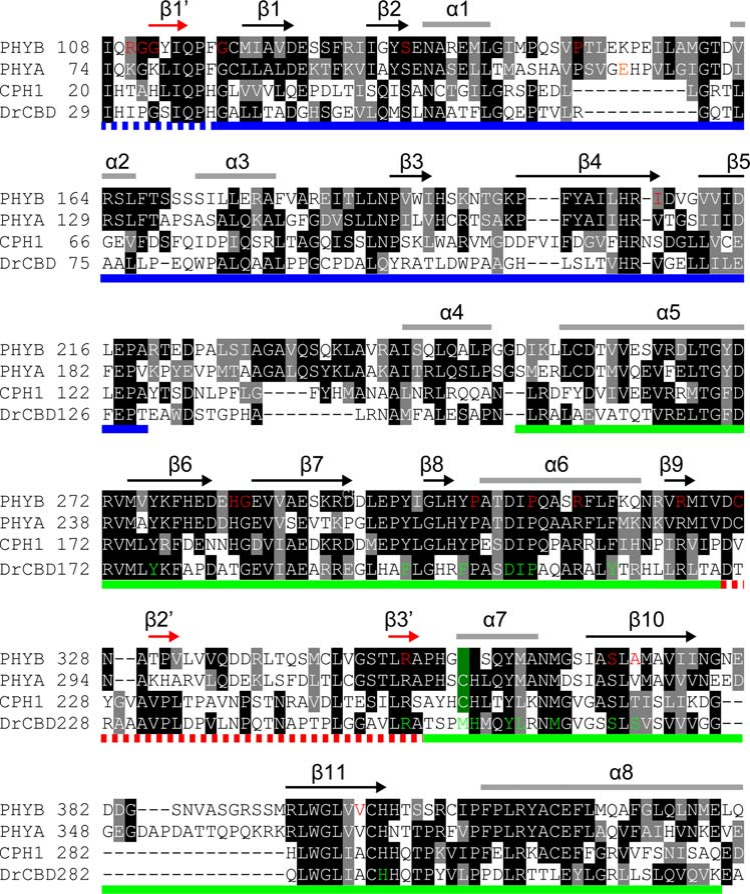
Alignment of *Arabidopsis* and Bacterial Phytochrome Sequences. The PLD-GAF region of phytochrome sequences are aligned. PHYB, *Arabidopsis thaliana* phyB; PHYA, *Arabidopsis thaliana* phyA; CPH1, *Synechosystis* PCC6803 Cph1; DrCBD, *Deinococcus radiodurans* BphP. Arrows and short bars on top of the sequences represent β-strands and α-helices, respectively. Blue and green lines at the bottom indicate PLD and GAF, respectively. Domains were delimited as for Figure 1A. A broken red line indicates the loop extended from GAF, which forms the light sensing knot together with the N-terminal end of PLD (broken blue). In the knot structure, the β1′, β2′ and β3′ strands (red arrows) form a small β-sheet. Amino acid residues at which mutations were found are indicated in red. The cysteine residues that bind the chromophore are indicated by green background. Amino acid residues that are in direct contact with the chromophore in *Dr*CBD are indicated in green. The three dimensional structure is based on [24].

Mutations D64N, R110Q, G111D, G112D, P149L, I208T, P304L and R352K reduced the biological activity of N651G-GUS-NLS without affecting the spectral properties *in vitro* (Table 1). Especially interesting are R110Q, G111D, G112D and R352K because alignment of the *Arabidopsis* phyB sequence with that of *Dr*CBD (Figure 5) suggested that these residues would reside in the vicinity of the light sensing knot (for detail, see discussion). Hence, we examined the biological activities of the full-length phyB carrying these mutations in transgenic *Arabidopsis*.

**Figure 6:**
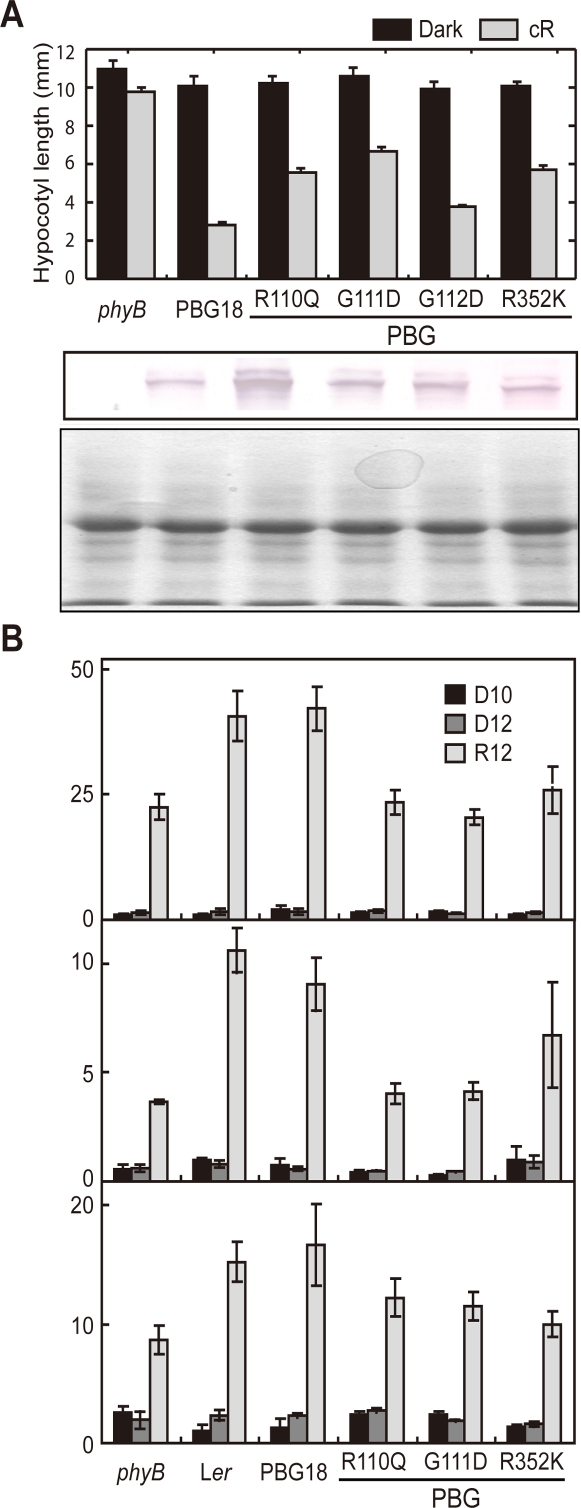
Regulatory Activity of Mutant Forms of PBG. (A) Hypocotyl lengths (upper panel) in transgenic *Arabidopsis* grown under cR (shaded) or darkness (closed) and immunoblot detection of PBG proteins (lower). For the hypocotyl measurements, plants were grown under cR (5.5 µmol m^−2^ sec^−1^) or in darkness for 5 days. Data are the mean±SE (*n* = 25). For the immunoblot detection of PBG proteins, 50 µg of total protein was loaded in each lane and PBG proteins were detected with a mouse monoclonal anti-phyB antibody (middle panel). To comfirm protein loading amount, the same samples were subjected to Coomassie Brilliant Blue (CBB) staining (lower panel). (B) Real-time PCR using RNA from 4-day-old seedlings grown in the dark (D0), kept in the dark for an additional 12 hr (D12) or exposed to cR for 12 hr (R12). Data are shown for *ELF4* (upper), *SAUR-LIKE* (middle) and *AMYLASE* (lower). Cycle threshold values were used to calculate fold-induction with Ler dark values set to 1. Values from three biological replicates are plotted with SE.

The mutated full-length phyB-GFP fusion proteins (PBG) carrying R110Q, G111D, G112D or R352K were expressed in the *phyB* mutant background under the control of the cauliflower mosaic virus 35S promoter. Immunoblot analysis revealed that the expression levels were comparable to or higher than those in PBG18 (Figure 6A). The long hypocotyl phenotype under cR was observed in PBG(R110Q), PBG(G111D) and PBG(R352K) mutants (Figure 6A). Exceptionally, the phenotype was less clear in PBG(G112D). This was probably due to the residual activity in this mutant. Indeed, the phenotype was weaker in the original N651(G112D)G-GUS-NLS mutant than the other 3 mutants (Figure 1B). It is not clear why these mutations showed weaker phenotypes in PBG background, compared to N651G-GUS-NLS background (Figure 1B). This was probably because of the fact that PBG line has a higher expression level than N651G-GUS-NLS line[20]. However, it is possible that the C-terminal moiety may acquire regulatory activity in conjunction with the photoactive N-terminal moiety, despite the observation that the C-terminal moiety alone does not show any apparent biological activity [20],[29].

Based on recent reports that early and late phases of phyB-regulated seedling deetiolation may involve different modes of regulation [38]–[40], we examined the effect of the R110Q, G111D and R352K mutations in the full-length PBG molecule on the cR-induced expression of three early-response genes, *ELF4* (*At2g40080*), *SAUR-LIKE* (*At4g38840*) and *AMYLASE* (*At4g17090*), shown previously, in time-course experiments, to be robustly phyB-dependent [11],[12],[41]. Twelve hr of cR exposure was selected for this experiment because the differential in expression between wild-type and *phyB*-null-mutant seedlings was found to be maximal at that time-point [11], providing maximal sensitivity for detecting reductions in cR sensitivity in our phyB-mutant variants. Although a small number of other genes had been reported to exhibit differences in expression at 1 hr of cR between the wild-type and *phyB*-null mutant by microarray analysis [12], none of these were found to display sufficiently robust differences at 1 hr cR by qPCR in our present analysis to permit reliable assessment of the effects of the point mutants identified here. Our data show that all three selected genes exhibit a similar pattern. Whereas the wild-type PBG sequence fully rescues the reduced cR-induced expression of the *phyB* mutant, all three mutant phyB variants fail to a greater or lesser extent to reinstate full induction of expression (Figure 6B). This pattern parallels the behavior of these variants in failing to complement the long-hypocotyl phenotype of the *phyB* mutant (Figure 6A), indicating a loss of phyB function in both early and late phases of the seedling deetiolation process.

### Subcellular Localization of Full-length phyB Mutant Variants

**Figure 7:**
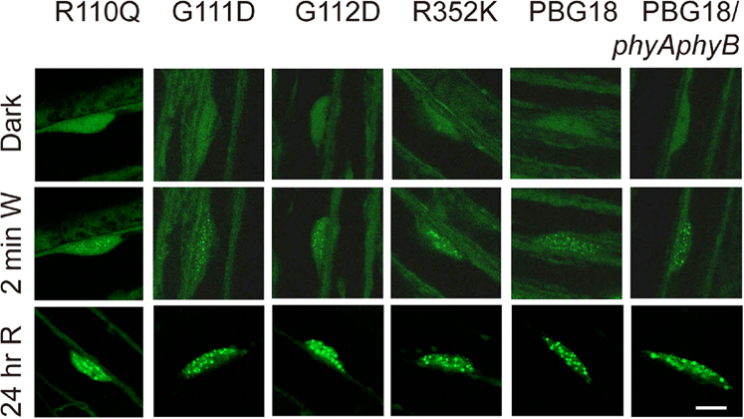
Subcellular Localization of Mutant Forms of PBG. Confocal microscopic observation of GFP fluorescence in transgenic *Arabidopsis* seedlings. Hypocotyl epidermal cells of 3-day-old seedlings were observed. Dark-grown seedlings (upper), those treated with cW for 2 min (middle) and those treated with cR for 24 hr (lower) are shown. The bar indicates 10 µm.

We confirmed that the intracellular localization of PBG was not affected by these mutations (Figure 7). The wild-type PBG as well as its mutated derivatives were detected not only in the cytoplasm but also in the nucleus in most of the cells in the etiolated seedlings. After 2 min irradiation with white light, early PBG speckles [42] were observed in the nuclear region in all derivatives. After 24 hr treatment with cR, nuclear accumulation and formation of late nuclear speckles were observed in all of the lines. The normal dynamics of these mutant PBG derivatives as regards subcellular localization indicates that these mutants are normal in photoperception. We also found that PBG formed both early and late speckles even on the *phyAphyB* double mutant background (Figure 7). Hence, formation of both early and late speckles was independent of the phyA function.

## Discussion

### Isolation and Classification of the Mutants

We recently demonstrated that phyB lacking a C-terminal moiety is still capable of robustly transducing a light signal to regulate normal seedling development [20]. Those results prompted us to elucidate the structural basis of this observation. Hence, we screened for long hypocotyl mutants to identify missense mutations that reduced the biological activity of phyB within the N-terminal domain of phyB. Prior to the present work, several missense mutations had been identified in phyA and phyB [23], [30]–[34],[43]. Of these, 8 mutations reside in the N-terminal moiety of phyB (Table 1), but the consequences of these residue substitutions to the molecular functions of the photoreceptor had only been examined for two of these. In the present study we identified 14 additional missense mutations and examined them in detail for functional relevance.

To identify as many novel mutations as possible, we modified the screening procedure, compared to previous studies. First, to focus on the N-terminal moiety of phyB, we used the N651G-GUS-NLS line as a parental line for mutagenesis. Second, the seedlings were grown under dim cR, which allowed us to detect smaller reductions in activity. Combined with a large scale screening of at least 1,000,000 M2 seedlings derived from 200,000 M1 plants, we successfully identified 14 novel missense mutations within the N-terminal moiety of phyB (Figure 1A and Table 1). It remains unclear why the present set of mutants did not overlap with the known ones. This might be because the N651G-GUS-NLS line rather than the full-length phyB line was used in the present study.

The 14 mutations found in the present study, together with the 8 previously described mutations [23], [31]–[34] were characterized with respect to their spectral properties *in vitro*, resulting in the identification of two principal classes of defects. One consists of the spectral mutants, which are defective in chromophore incorporation, photoconversion and/or stability of Pfr. The other comprises signaling mutants, which are normal in spectral properties but defective in biological activity. 14 mutations out of the total of 22 were classified as spectral mutants and the remaining 8 as signaling mutants.

As the loss of spectral integrity directly affects the amount or overall structure of the active Pfr form of the photoreceptor, the reduced biological activity of the spectral mutants is simply explained by the low amount of or aberrant Pfr form. These mutants are, therefore, defective in normal light signal perception. It is well established that mutation at the chromophore attachment site (C357S of phyB), preventing chromophore ligation, shows loss of biological activity [29], and the N-terminal 450 amino acid-fragment of phyB which exhibits an aberrant Pfr form and fast dark reversion has reduced biological activity [23]. Of the fourteen mutants newly studied here, seven (G284E, P309L, R313K, R322Q, S370F, V401L and S584F) are photoperception mutants. Of these, two (P309L and S370F) display essentially complete loss of photosensory activity in vivo (Figure 1B), consistent with the absence or severe loss of chromophore ligation capacity (Figure 2), whereas the remainder display reduced photosensory activity, consistent with varying degrees of spectral aberration (Figures 3 and 4).

By contrast, the remaining seven of the fourteen mutants studied here (D64N, R110Q, G111D, G112D, P149L, P304L and R352K) retain spectral integrity (Figures 2,3 and 4), indicating that they are normal in light signal perception, but defective in signal transfer to downstream components of the phyB transduction chain. The retention of normal spectral properties by these mutant molecules is a strong indication that they retain the broad structural integrity of the N-terminal moiety, because of the well-established evidence that deletion of any of the major subdomains causes aberrant spectral properties and altered biological activity [44]. In addition, of the four signaling mutants examined here in the context of the full-length phyB protein, all showed nuclear localization and normal intranuclear dynamics upon light activation (Figure 7). This result strongly indicates that these mutations specifically disturb the signal transferring function without reducing other functions of phyB.

It is notable that of the nine phyB-variant lines showing an intermediate phenotype (intermediate hypocotyl length between the *phyB* and the N651G-GUS-NLS transgenic rescue lines), six (G248E, P304L, R313K, R322Q, V401L and S584F) show some degree of reduced responsiveness to the lower compared to the higher intensity. With the exception of P304L, these are all photoperception mutants, compromised in their spectral activity, consistent with the prediction that they will have reduced photosensory sensitivity. The remaining three (G112D, P149L and R352K) do not show such a difference in hypocotyl responsiveness to the cR intensity. This is also not unexpected, because these are signal-transfer mutants. These exhibit normal photoperception, but reduced regulatory activity in inhibiting hypocotyl elongation. This behavior is consistent with the prediction that these mutants will retain the same equal sensitivity as the parent N651G-GUS-NLS molecule to the two cR intensities, but have reduced capacity to transduce the perceived light signal (this second step being independent of the intensity of the signal at saturation).

### Overall Distribution Pattern of the Mutations in the N-terminal Moiety

All 22 mutations were mapped within the phyB amino acid sequence (Figure 1A). These mutations were more or less evenly distributed throughout the N651 moiety, suggesting that all subdomains are important for the normal function of N651. However, the different types of mutations distributed differently. The spectral mutations are distributed mainly in the GAF and PHY subdomains (Table 1). By contrast, the signaling mutations tend to cluster in both the N-terminal extension and PLD. This observation thus defines the roles of the subdomains in the N-terminal moiety: GAF and PHY are apparently responsible for light-signal input (photoperception and/or maintenance of the Pfr form), whereas the N-terminal extension and PLD are mainly involved in signal transduction by phyB. This conclusion is consistent with the fact that GAF forms the chromophore pocket [24] and PHY stabilizes phyB in the Pfr form [23]. Similarly, it has been shown that deletion of the N-terminal extension reduces the biological activity of phyB [29]. Although the importance of PLD to the signal transfer function of phytochrome has not been reported, many PAS domains are known to be involved in protein-protein interactions [45], implying that PLD may be directly involved in the interaction with downstream signaling components such as PIF3 [13]–[15].

Recently, the three dimensional structure of the bacterial phy *Dr*CBD has been determined [24]. The data show that the PAS domain of *Dr*CBD exhibits a typical PAS fold while the GAF domain constitutes the chromophore-binding pocket in which the phytochromobilin chromophore is buried. Especially interesting is an unusual three dimensional structure, the proposed “light sensing knot”, found between the PAS and GAF domains. Alignment of the phyB sequence with that of *Dr*CBD allowed us to predict the positions of the mutated residues in the three dimensional model (Figure 5). The chromophore is surrounded by a β-sheet consisting of β6-11 strands and two α-helices (α6 and 7) in the *Dr*CBD chromophore pocket [24]. All of the mutations in the GAF domains except R352K were predicted to be within this region (Figure 5). These amino acid residues are highly conserved among diverse phytochromes. Of the PLD mutations, R110Q, G111D and G112D were predicted to be within or in the vicinity of the β1′-strand, which is one of the partners for β3′ in formation of the knot [24]. G118R, S134G, P149L and I208T were located between β1′ and β1, at the end of β2, between α1 and α2, and at the end of β4, respectively (Figure 5). These amino acid residues mutated in PLD are, for the most part, not highly conserved among phytochromes, with the exception of G118 and S134, which reduce the chromophore incorporation.

### Light-Signal Perception Mutants

We employed an *in vitro* reconstitution system [37] to examine the spectral properties of mutant N651 derivatives. Zn-blot analysis effectively identified mutants that were deficient in chromophore incorporation (Figure 2). Chromophore incorporation was severely impaired in the G118R, S134G and S370F mutants. In addition, reduced chromophore incorporation was observed in G284E and P309L, both of which also exhibited abnormal difference spectra (Figure 3). In another subclass of mutants, which included R322Q, A372T and S584F, chromophore incorporation was normal but the difference spectrum was altered (Figure 3).

Alignment of the phyB sequence with that of *Dr*CBD allowed us to predict the positions of the mutated residues in the three dimensional model (Figure 5). In the following description, amino acid residues in *Dr*CBD are shown in parentheses. As expected, many of the chromophore incorporation and difference spectrum mutations mapped to the vicinity of the chromophore. Indeed, close interactions of S370(S272) and A372(S274) with the chromophore in *Dr*CBD has been reported [24] (Figure 5). In addition, G284(G184), P309(P209) and R322(R222) are situated in the vicinity of the chromophore.

It remains unclear why mutations in G118(G39)R and S134(S55)G severely disturbed chromophore incorporation. These residues reside in PLD. In the three dimensional model, these residues are spatially separated from the chromophore pocket in *Dr*CBD [24] (Figure 5). However, there are reports that indicate the involvement of PLD in chromophore incorporation. The N-terminal 225 amino acid deletion abolishes chromophore incorporation in *Arabidopsis* phyA[46]. The I80 residue of pea phyA, which corresponds to I114(I35) of *Arabidopsis* phyB, is critical for chromophore binding [47]. Insight into the means by which these residues in PLD contribute to chromophore binding awaits elucidation of the three dimensional structure of higher plant phytochrome.

### Pfr Stability Mutants

The dark reversion rate, which reflects the stability of Pfr in darkness, is an important process regulating the level of Pfr *in vivo*. Mutants defective in Pfr stability are thus compromised in normal light-signal perception. Indeed, a faster dark reversion rate has been shown to reduce the physiological activity of phyB [23]. We observed faster dark reversion in 9 of 17 mutants examined (Figure 4A, Table 1). It is known that PHY stabilizes Pfr [22],[23],[48]. Concordantly, each of the three PHY mutants (G564A, S584F, A587T) produced higher dark reversion rates.

The other mutants that exhibited faster dark reversion (H283T, R313K, R322Q, C327Y, A372T, V401I) were found to be mutations in GAF. This is not surprising because GAF constitutes the chromophore binding pocket [24]. In the *Dr*CBD three dimensional structure, A372(S274) directly interacts with the C-ring of the chromophore molecule. In addition, R322(R222) and V401(A288) reside in the chromophore pocket. H283(T183), R313(R213) and C327(T227) are a little more distant but still in the vicinity of the chromophore pocket.

### Signaling Mutants

Including I208T identified in a previous study [32], eight mutants that exhibited reduced biological activity with no effect on spectral activity are defined as signaling mutants (Table 1). One mutation (D64N) was found in the N-terminal extension consistent with the reports that, although no structural information is yet available, the N-terminal extension is important for the signal transduction activity of phyB [29]. Two mutations, P304L and R352K, were found in GAF. R352 is particularly interesting because it is presumed to reside in the vicinity of both the chromophore and the light sensing knot (see below). The reason why P304L reduced the signaling activity is less clear. However, P304(P204) is next to Y303(F203), which interacts with ring D of the chromophore in *Dr*CBD [24] suggesting that P304(P204) might affect signaling activity through an interaction with the ring D.

The other 5 signaling mutants were found in PLD, suggesting that this domain is important for the signal transduction activity. Particularly interesting are the three successive mutations, R110Q, G111D and G112D. Interestingly, R110(I31), G111(P32) and G112(G33) partly overlap with the β1′ strand which, together with the β2′ and β3′ strands in *Dr*CBD, participates in the formation of the light sensing knot [24] (Figure 5). Hence, the present data are consistent with the idea that the light sensing knot plays a critical role in phytochrome signal transduction. Two additional mutants, P149L and I208T, were found in PLD. The I208(V118) residue is at the end of the β4 strands and faces the knot in the *Dr*CBD structure [24]. The P149(R70) reside is in the loop connecting the α1 and α2 helices and faces the knot as well.

The R352(R254) residue forms salt bridges through its two amines with the carbonyl oxygen of the ring B propionate of the chromophore in *Dr*CBD [24]. Since one of these amines is missing in the R352K mutant, the mutation would be expected to weaken the interaction between ring B and the polypeptide moiety. Because of the tight connection with the chromophore, the R352K mutant might be expected to have negatively affected photochemical properties. Indeed, the substitution to E of R318 in pea phyA, and that to K of R254 in cph1, which correspond to R352 of *Arabidopsis* phyB, altered their photochemical property [49],[50]. Nevertheless, abnormal spectral properties of R352K were less clear in the N-terminal moiety of phyB (Figures 2–4). This may be because of the different phytochrome species involved. Furthermore, PBG(R352K) accumulated in the nucleus and formed speckles in a light-dependent manner (Figure 7), which strongly indicates that PBG(R352K) was spectrally active *in vivo*.

One surprising feature of R352(R254) is its proximity to the light sensing knot. In the *D*rCBD structure, R352(R254) is on the β3′ strand, which is a component of the knot (Figure 5). The three successive R110(I31), G111(P32) and G112(G33) residues are partly included in β1′, which is one of the partners for β3′ in formation of the knot [24]. Considering the possible tight connection of R352(R254) with the chromophore, these four amino acid residues may constitute a route to relay the conformational changes in the chromophore to the surface of the molecule. It should be noted here that the model presented here is based on the *D*rCBD structure. Unfortunately, the homology is not particularly high between higher plant phyB and *Dr*CBD within PLD (Figure 5). Consequently, the three dimensional structure of phyB may be different from that of *Dr*CBD. To answer the question definitively, the three dimensional structure of phyB needs to be determined.

It is notable, that the disruption of the signal transfer capacity of the phyB molecule by mutations in the light-sensing knot region have parallel deleterious effects on both early and late phases of seedling deetiolation regulated by phyB. This suggests that these amino acids have a central role in the primary signaling function of the photoreceptor molecule.

## Materials and Methods

### Plant Materials, Growth Conditions for Seedlings, and Growth Measurements

The *Arabidopsis thaliana* mutant, *phyB-5*, is a null allele on the Landsberg *erecta* background [34]. The PBG [9] and N651G-GUS-NLS (originally NG-GUS-NLS) [20] lines on the *phyB-5* background and the PBG18 line on the *phyA-201phyB-5* double mutant background [23] have been described elsewhere.

Seeds were surface-sterilized and sown on 0.6% agar plates containing Murashige-Skoog (MS) medium with or without 2% (w/v) sucrose. The plates were kept in the dark at 4°C for 72 hr and then irradiated with continuous white light (cW) for 3 hr at 22°C to induce germination. The plates were then placed under various light conditions, as specified in the figure legends. The light sources were as described previously [23]. For hypocotyl length measurements, the seedlings were grown on MS agar plates without sucrose for 5 days at 22°C and then pressed gently onto the surface of agar medium before photographs were taken. Hypocotyl length was determined by the NIH image software (Bethesda, ND). For immunoblot analysis, the seedlings were grown on MS agar plates with 2% (w/v) sucrose for 1 week at 22°C in cW (45 µmol m^−2^ sec^−1^).

### EMS Mutagenesis and Screening

Seeds of the N651G-GUS-NLS expressing *Arabidopsis* line, 4-1, were mutagenized with 0.3% EMS. Approximately 600 seeds were sown directly onto soil in individual pots. Growth in each pot, which consisted of about 300 plants, was considered an M1 family. From each M1 family, M2 seeds were collected. One to two thousand M2 seeds were then subjected to screening. Seedlings were screened visually for tall phenotype after 5 days under weak cR (0.05 µmol m^−2^ sec^−1^). M3 seedlings were then examined for hypocotyl lengths in weak cR and cFR (10 µmol m^−2^ sec^−1^). Lines that were taller only in cR were backcrossed to the *phyB* mutant. The long hypocotyl phenotype was examined in both F1 and F2 generations to determine if the mutation was linked to the *N651G-GUS-NLS* locus. The light sources employed have been described elsewhere [23].

### Sequence Analysis of Mutants

Crude plant DNA was prepared from the M3 plants. The *N651* fragment of *N651G-GUS-NLS* was amplified using PCR primers complementary to the cauliflower mosaic virus 35S promoter and GFP regions. Purified PCR products were sequenced using BigDyeTerminator V3.1 Cycle Sequencing Kit (Applied Biosystems).

### Immunochemical Experiments

Protein extraction, SDS-polyacrylamide gel electrophoresis, protein blotting, and immunodetection were performed as described [9]. Antibodies used were a monoclonal anti-phyB mBA1 antibody [51], an anti-GFP monoclonal antibody (SIGMA) and antiserum against chitin binding domain (New England Biolabs).

### *Escherichia coli* Expression and Reconstitution

For N651 protein expression, the N651 fragment was cloned into the pTYB2 vector containing Intein/CBD (New England Biolabs) [23]. Mutations were introduced into N651 using the QuikChange Site-Directed Mutagenesis Kit (Stratagene). *Escherichia coli* transformation and expression of wild type and mutant N651-Intein/CBD fusion proteins were performed as previously described [23]. Intact holoproteins were reconstructed using PCB as a chromophore [37]. The resultant holoproteins were subjected to spectral analyses.

### Spectrophotometric Assays

The Zn blot, difference spectra, and dark reversion analyses were essentially as described previously [23]. For Zn blot analysis, extracts containing equal amounts of N651-Intein/CBD protein were loaded onto the gel. To ensure equal sample loading, immunodetection of Intein/CBD fusion proteins was performed in advance.

### Plasmid Construction and Plant Transformation

To generate mutant *PBG* constructs, mutations were introduced into *PBG* using the QuikChange Site-Directed Mutagenesis Kit (Stratagene). Mutant *PBG*s were inserted between the cauliflower mosaic virus 35S promoter and the Nos terminator of pPZP211/35S-nosT, which is itself derived from pPZP211 [52]. The *phyB-5* mutant was used as the host for transformation by the *Agrobacterium*-mediated floral dip method [53]. Transformed plants were selected on MS medium containing 25 µg mL^−1^ kanamycin and 166 µg mL^−1^ claforan (Hoechst) and by microscopic observation of GFP fluorescence.

### Gene Expression Analysis

RNA isolation, cDNA synthesis and the real-time PCR were performed essentially as described [38]. The specific primer sequences were as follows: *ELF4*-F, 5′-CGACAATCACCAATCGAGAATG-3′, *ELF4*-R, 5′-AATGTTTCCGTTGAGTTCTTGAATC-3′, *SAUR-like*-F, 5′-TTCTTCACTGCAAGGGATTGTG-3′ *SAUR-like*-R, 5′-AAAGGCAGAGGAAGAGTTTGGA-3′ *AMYLASE*-F, 5′-AAAGCACGGTCTCAAACTCC-3′, and *AMYLASE*-R, 5′-CACAGAATCACATCCCAAGG-3′. The gene *PP2A* (*At1g13320*), which is expressed at similar level in darkness or red light (data not shown), was used as a normalization control [54]. Each PCR was repeated three times. Gene expression data were represented relative to the average value for the wild type grown in darkness in each experiment, after normalization to the control. The experiment was performed with three independent biological replicates.

### Analysis of Subcellular Localization in Transgenic *Arabidopsis* Seedlings

Seedlings were grown on MS agar plates without sucrose for 3 days at 22°C in darkness. Seedlings were set on the stage of a confocal laser microscope (Olympus) and nuclei were located under green safe light by conventional microscopic observation. Seedlings were scanned once to observe GFP fluorescence [9] and then irradiated with the microscope white lamp for 2 min. After irradiation, the seedlings were scanned again. For long-term irradiation, seedlings were treated with cR of 44 µmol m^−2^ sec^−1^ for 24 hr.
